# Data integration by multi-tuning parameter elastic net regression

**DOI:** 10.1186/s12859-018-2401-1

**Published:** 2018-10-10

**Authors:** Jie Liu, Gangning Liang, Kimberly D Siegmund, Juan Pablo Lewinger

**Affiliations:** 1 Department of Preventive Medicine, USC Keck School of Medicine, 2001 N Soto Street, Los Angeles, CA 90089 USA; 2 USC Institute of Urology and the Catherine & Joseph Aresty Department of Urology, Norris Comprehensive Cancer Center, University of Southern California, Los Angeles, CA 90089 USA

**Keywords:** Data integration, Classification, Elastic net

## Abstract

**Background:**

To integrate molecular features from multiple high-throughput platforms in prediction, a regression model that penalizes features from all platforms equally is commonly used. However, data from different platforms are likely to differ in effect sizes, the proportion of predictive features, and correlations structures. Subtle but important features may be missed by shrinking all features equally.

**Results:**

We propose an Elastic net (EN) model with *separate* tuning parameter penalties for each platform that is fit using standard software. In a comprehensive simulation study, we evaluated the performance of EN logistic regression with multiple tuning penalties. We found that when the number of informative features differs among the platforms, and when there is no notable correlation between the features from different platforms, the multi-tuning parameter EN yields more predictive models. Moreover, the multi-tuning parameter EN is robust, in the sense that there is no loss of predictivity relative to a single tuning parameter EN when features across all platforms have similar effects. We also investigated the performance of multi-tuning parameter EN using real cancer datasets.

**Conclusion:**

The proposed multi-tuning parameter EN model, fit using standard penalized regression software, can achieve better prediction in sample classification when integrating multiple genomic platforms, compared to the traditional method where a single penalty parameter is used for all features in different platforms.

**Electronic supplementary material:**

The online version of this article (10.1186/s12859-018-2401-1) contains supplementary material, which is available to authorized users.

## Background

As multi-platform profiling of tissues enabled by advances in high-throughput ‘omic’ technologies becomes routine, efficient statistical methods to integrate multi-omic data is becoming increasingly important. Multi-omic profiling has been used to successfully investigate prognostic biomarkers and identify aberrant pathways in cancer [1, 2], enhance clustering and subclassification [3, 4], and improve prediction of cancer prognosis and therapeutic response [5–7]. Multi-omic data integration can be challenging for several reasons. First, different data types will typically have different scales of measurement. To meaningfully integrate data sets with diverse scales, proper standardization and/or data transformation is required. A second challenge is the increasingly high-dimensionality of multi-platform ‘omic’ data. This could be addressed by feature pre-screening methods such as sure independence screening [8], or by dimensional reduction techniques such as principal component analysis [9] or partial least squares [10]. The third challenge, which to the best of our knowledge has not been yet fully addressed, is the potentially different contributions of individual data types in the final prediction models.

Regularized regression with a sparsity inducing penalty (e.g. LASSO [11], Elastic Net [12], SCAD [13]) is a common approach to feature selection for building predictive models based on high-dimensional data, and can be effectively used for a joint analysis of multi-omic profiles measured on the same samples. For example, Taskesen et al. used the standard LASSO for classification of samples measured on methylation and expression platforms [14] by including all the features from both platforms. In regularized regression, a tuning parameter controls the degree of shrinkage applied to the regression coefficients, and penalties that induce sparsity shrink many coefficients to exactly zero, performing in effect model selection. However, typical regularized regression approaches, would apply the same degree of shrinkage to all features regardless of their omic type, which can be suboptimal if the number and effect sizes of predictive features differ between data types. If for example, there were fewer predictive gene expression features than DNA methylation features, but the predictive expression features had larger effects than the predictive methylation features, forcing a common degree of shrinkage could result in all methylation feature coefficients shrunk to zero, and a final model containing only expression features. Independently predictive methylation features with subtler effects would be missed. Approaches that account for these differences may offer improved predictive performance.

A natural question of interest is whether allowing for differential shrinkage across features from different omic types by using a separate tuning parameter for each type, can yield better predictive models. Taskesen et al. addressed this to some extent in a stage-wise analysis, performing separate analysis of omic platforms prior to creating a single classifier from the posterior probabilities of each model fit [14]. In this approach, individual features from the separate platforms were not combined in their final classifier but only used to pick the more predictive data type. Even if the selected features from multiple platforms were combined, such a two-stage approach would likely result in a different set of selected features having different prediction potential. It is this joint analysis using features from multiple platforms that is our goal. Note that allowing differential shrinkage across different platforms is a distinct issue from how to deal with subgroups of features, such as co-regulated genes within a cluster structure, that one may have reason to believe a-priori are either all predictive or not as a group, and which has been addressed by methods such as the group LASSO [15–17] that encourage the selection of either all or none of the features in each subgroup.

In this paper, we investigate the performance of a multi-tuning parameter elastic net regression (MTP EN) with separate tuning parameters for each omic type. Through simulations with a range of scenarios differing in number of predictive features, effect sizes, and correlation structures between omic types, we show that MTP EN can yield models with better prediction performance. We apply the MTP EN to publically available prostate cancer and acute myeloid leukemia data sets from the Cancer Genome Atlas (TCGA) and Gene Expression Omnibus (GEO).

## Methods

### Setup and notation

We assume we have training data consisting of high-dimensional features from multiple omic types (e.g. gene expression, DNA methylation, somatic mutations, etc.), measured on *n* samples, and an outcome of interest (e.g. cancer vs. normal tissue). The goal is to use the training data to build a model that can be used to predict the outcome for new samples based on their corresponding multi-omic profiles. This is a standard supervised learning setting with the particularity that the features are from multiple omic types.

For simplicity, we focus our presentation on a binary outcome and omic features of two types, but the multi-tuning parameter regression framework can be applied to other outcomes (e.g. multinomial, continuous, time to event, etc.) and more than two data types. For sample *i*, *i* = 1, …, n, we denote the binary outcome by *y*_*i*_ ∈ {0, 1} and its corresponding omic profile as the partitioned vector ${\boldsymbol{x}}_{\boldsymbol{i}}=\left({\boldsymbol{x}}_i^{(1)},{\boldsymbol{x}}_i^{(2)}\right)$, where ${\boldsymbol{x}}_i^{(1)}$ and ${\boldsymbol{x}}_i^{(2)}$ represent the features from the first and second omic type, respectively. The number of features of each type is usually much larger than the sample size *n*, and is in the hundreds or thousands in typical studies. For example, gene expression is measured for thousands of genes/features, DNA methylation for hundreds of thousands of CpGs, and genotypes for millions of SNPs. We let *p*_1_ and *p*_2_ be the number of features of the first and second omic type respectively, and *p* = *p*_1_ + *p*_2_ be the total number of features. In typical applications, there is also a low-dimensional vector of personal and/or clinical features (e.g. age, gender, etc.), that may also be predictive and included in the feature set, but for simplicity we omit here any additional non-omic features.

We denote by ***y =*** (*y*_1_, …, *y*_*n*_) the vector of outcomes and by **X**^(1)^ and **X**^(2)^ the design matrices whose rows are the *i*^th^ sample vector ${\boldsymbol{x}}_i^{(1)}$ and ${\boldsymbol{x}}_i^{(2)}$ defined above. We denote by **X**= [**X**^(1)^| **X**^(2)^] the matrix containing the full set of features obtained by row-wise concatenation of **X**^(1)^ and **X**^(2)^.

In addition to constructing a model that generalizes well, i.e. accurately predicts ***y*** based on ***X*** in new subjects, a parsimonious prediction model based on a small subset of the full feature set is generally preferred. A model with fewer features is more interpretable and more easily translated into a custom assay deployable in a clinical setting.

Regularized regression with a sparsity inducing penalty is an effective way to simultaneously perform feature selection and parameter estimation to build a predictive model with a small subset of features. The simplest and most commonly used sparsity inducing penalty is the L_1_ norm, which gives rise to LASSO regression [11]. Regularization with a combination of the L_1_ and L_2_ norms, i.e. elastic net regression, typically outperforms feature selection with the LASSO in settings with correlated features [18]. Many additional extensions and variations of the LASSO have been proposed to, for example, reduce over-shrinkage of larger coefficients (adaptive LASSO [19]) or to handle features with additional structure (e.g. group LASSO [15], fused LASSO [20], group LASSO with overlap [21], graph LASSO [22]). For definiteness, in this paper we focus on the elastic net, which includes the LASSO as a special case. However, customized tuning parameters for each omic type can be used with any type of regularized regression.

The elastic net regularization penalty is a weighted mixture of the LASSO (L_1_ norm) and ridge (square of L_2_ norm) penalties given by: $N\left(\boldsymbol{\beta} \right)=\left(1-\alpha \right)\frac{\mathbf{1}}{\mathbf{2}}{\left\Vert \boldsymbol{\beta} \right\Vert}_{{\boldsymbol{L}}_{\mathbf{2}}}^{\mathbf{2}}+\alpha\ {\left\Vert \boldsymbol{\beta} \right\Vert}_{{\boldsymbol{L}}_{\mathbf{1}}}=\left(1-\alpha \right)\frac{\mathbf{1}}{\mathbf{2}}\sum \limits_{j=1}^p{\beta}_j^2+\alpha \sum \limits_{j=1}^p\left|{\beta}_j\right|$, where *α*, 0 ≤ *α* ≤ 1, is the weight given to the LASSO penalty and 1 − *α* the weight given to the ridge penalty. Both the LASSO (*α* = 1) and the ridge (*α* = 0) penalties are particular cases of the elastic net penalty.

Standard elastic-net logistic regression solves the penalized regression problem given by:

1 $$ {\min}_{\boldsymbol{\beta} \in {\mathrm{\mathbb{R}}}^p}-l\left(\boldsymbol{y},\boldsymbol{X};{\beta}_0,\boldsymbol{X}\boldsymbol{\beta } \right)+\uplambda N\left(\boldsymbol{\beta} \right) $$

where $l\left(\boldsymbol{y},\boldsymbol{X};{\beta}_0,\boldsymbol{\beta} \right)={\sum}_{i=1}^n\log \left(1+\exp \left({\beta}_0+{\boldsymbol{x}}_i^T\boldsymbol{\beta} \right)\right)-{\sum}_{i=1}^n{y}_i\left({\beta}_0+{\boldsymbol{x}}_i^T\boldsymbol{\beta} \right)$ is the standard logistic log-likelihood function and the regularization parameter λ ≥ 0 controls the degree of penalization applied to the vector of regression coefficients ***β*** (except the intercept *β*_0_ which is typically not penalized). The single regularization parameter λ is common to all features and is usually tuned by cross-validation.

In this paper, we propose using separate tuning parameters for each omic type by solving the penalized regression problem given by,

2 $$ {\min}_{\boldsymbol{\beta} \in {\mathrm{\mathbb{R}}}^p}-l\left(\boldsymbol{y},\boldsymbol{X},{\beta}_0;\boldsymbol{\beta} \right)+{\uplambda}_1N\left({\boldsymbol{\beta}}^{(1)}\right)+{\uplambda}_2N\left({\boldsymbol{\beta}}^{(2)}\right) $$

where the vector of regression coefficients ***β =*** (***β***^(1)^, ***β***^(2)^) is partitioned according to the omic type conformably to ***X =*** [**X**^(1)^| **X**^(2)^]***.***The regularization parameters λ_1_ ≥ 0 and λ_2_ ≥ 0 are now specific to each omic type. Our hypothesis is that a ‘custom’ degree of regularization for each type can account for intrinsic differences between the data types and lead to a selected model with better prediction performance.

#### Model fitting and parameter tuning

Although the two-tuning parameter model (2) is non-standard, it can be fitted using elastic-net regression software provided it has an option to use a weighted elastic-net penalty of the form

${N}_{\boldsymbol{w}}\left(\boldsymbol{\beta} \right)=\alpha {\sum}_{j=1}^p{w}_j\left|{\beta}_j\right|+\left(1-\alpha \right){\sum}_{j=1}^p{w}_j{\beta}_j^2$. Using a weighted penalty, the multi-tuning parameter elastic-net penalty in (2) can be equivalently written as

$$ {\uplambda}_1N\left({\boldsymbol{\beta}}^{(1)}\right)+{\uplambda}_2N\left({\boldsymbol{\beta}}^{(2)}\right)={\uplambda}_1{N}_{\boldsymbol{w}}\left(\boldsymbol{\beta} \right) $$

with a *p*-dimensional weight vector $\boldsymbol{w}=\left(1,1,\dots, 1,\frac{\uplambda_2}{\uplambda_1},\frac{\uplambda_2}{\uplambda_1},\frac{\uplambda_2}{\uplambda_1}\right)$ with 1 in its first *p*_1_ entries (corresponding to the *p*_1_ features of the first omic type and the dimension of ***β***^(1)^) and a $\kappa =\frac{\uplambda_2}{\uplambda_1}$in the next *p*_2_ entries (corresponding to the *p*_2_ features of the second omic type and the dimension of ***β***^(2)^). Thus, the two-penalty model can be alternatively specified using the tuning parameters λ = λ_1_, $\kappa =\frac{\uplambda_2}{\uplambda_1}$, where λ controls the overall shrinkage on both types of omic features, and *κ* controls the shrinkage ratio between the two types. For *κ* = 1, the model reduces to the standard elastic net with a single tuning parameter.

To investigate the performance of the multi-tuning parameter elastic net regression (MTP EN) for building predictive models based on multi-omic features we conduct a series of simulations under a range of scenarios. To fit the MTP EN we use the efficient and widely used elastic-net implementation in the glmnet R package [23], which allows for a user-specified weighted penalty via the “*penalty.factor*” argument. Additional file 1 contains an R script implementing a complete and self-contained analysis example using MTP EN.

We set the elastic net penalty *α* to ½ and tune the overall shrinkage, λ, and shrinkage ratio parameter, *κ*,by k-fold cross-validation (CV), with the area under the ROC curve, AUC, as the performance metric. The training data is randomly split into *k* equally-sized folds, each with (approximately) the same proportion of positive (*y* = 1) and negative (*y* = 0) samples as the full training data. The MTP EN is trained on *k* − 1 folds for all values of (λ, *κ*) in a grid of possible tuning parameter values and the AUC is computed based on the validation held-off fold. This is repeated, using in turn each of the folds as validation held-off fold. The ‘optimal’ tuning parameters are the values *κ* = *κ*_*max*_ and λ = λ_*max*_ that maximize the average AUC across all folds. The optimal tuning parameter values are then applied to a fully independent test set to unbiasedly assess the model AUC. For model tuning we utilized both 5-fold and 10-fold CV, 5-fold for the analysis of real data because of a small sample size in one class and 10-fold for the simulation study.

### Simulation study

We evaluate the MTP elastic-net through simulation, exploring varying proportions and effect sizes of the relevant features in each omic type, varying dimensionalities of the feature sets, and a range of correlation structures. Specifically, the binary outcome was generated based on a logistic regression model of the form:

$$ {y}_i\mid {\boldsymbol{x}}_i^{(1)},{\boldsymbol{x}}_i^{(2)}\sim Bernoulli\left({P}_i\right) $$

$$ {P}_i=\frac{\exp \left(\eta \right)}{1+\exp \left(\eta \right)} $$

$$ {\eta}_i={\beta}_0+{\boldsymbol{x}}_i^{\left(\mathbf{1}\right)\boldsymbol{T}}{\boldsymbol{\beta}}_{\left(\mathbf{1}\right)}+{\boldsymbol{x}}_i^{\left(\mathbf{2}\right)\boldsymbol{T}}{\boldsymbol{\beta}}_{\left(\mathbf{2}\right)} $$

where we set *q*_*j*_ features among the *p*_*j*_ in omic type *j* = 1, 2 (*q*_*j*_ ≪ *p*_*j*_) to be predictive by arranging the vector of regression coefficients ***β***_(***j***)_ to be sparse, with *q*_*j*_ non-zero and *p*_*j*_ − *q*_*j*_ zero entries. We set the effect size of the predictive features, i.e. the non-zero entries in omic type *j* = 1, 2 to a common value *β*_*j*_.

We generated feature data ***X =*** [**X**^(1)^| **X**^(2)^] by sampling from a multivariate normal distribution ***X~N***(**0**, **Σ**), where **Σ** is a population covariance matrix with the following structure: i) *dia*(**Σ**) ***=*** **1**, i.e. the ***X***’s are already standardized and have marginal variances equal to one; ii) *r*_*j*_ features among the *q*_*j*_ informative ones in omic type *j* = 1, 2 have a common pairwise correlation *ρ*_*j*_ and correlation *ρ*_12_ with the counterpart set of features in the other platform iii) all remaining correlations are zero. The simulation scenarios are summarized in Table 1. For each scenario, we simulated 400 replicate data sets, 200 training and 200 test sets. Every data set included 500 features on 200 samples, with an expected 100 samples in each class (*β*_0_ = 0).

**Table 1:** Summary of simulation scenarios

Independent Features*: ρ*_1_ = *ρ*_2_ = *ρ*_12_ = 0					
Scenario #	*β*_1_	*β*_2_	*q*_1_	*q*_2_	Optimal penalty ratio (κ^*^)
1	0.6	0.8	5	20	0.55
2	0.6	0.6	5	20	0.70
3	0.8	0.6	5	20	0.75
4	0.8	0.8	5	5	1
5	0.8	0.6	5	5	1
Correlated Features: *β*_1_ = 0.8, *β*_2_ = 0.6, *q*_1_ = 5, *q*_2_ = 20, *r*_1_ = *r*_2_ = 3					
	*ρ*_1_	*ρ*_2_	*ρ*_12_	Optimal penalty ratio (*κ*^∗^)	
6	0.4	0.2	0	0.85	
7	0.4	0.2	0.4	0.9	

Two hundred samples per data set, 250 features per omic type, 2 omic types. The performance of MTP EN is evaluated by varying effect sizes, number of informative features, and correlation structures between omic types. Specifically, *ρ*_1_ is the correlation between informative features in platform 1, *ρ*_2_ is the correlation between informative features in platform 2, *ρ*_12_ is the correlation between informative features from the different platforms, *β*_1_ and *β*_2_ are the effect sizes of informative features in platforms 1 and 2, respectively, while *q*_1_ and *q*_2_ are the numbers of informative features

Model tuning parameters (λ, *κ*) were selected using the training data, and applied to the test data set for estimating prediction performance. To further reduce the dimensionality of the selected features beyond what is achieved by maximizing the training data prediction performance, we set λ to λ_1*se*_, the largest value of λ such that the AUC is within one standard error of the maximum (achieved at λ = λ_*max*_). This strategy by Friedman et al. [12] yields predictive performance similar to that achieved by setting λ = λ_*max*_ but with a more parsimonious model. Thus, the parameters *κ* = *κ*_*max*_ and λ = λ_1*se*_, are used to estimate AUC from the independent test data set.

In addition to the AUC, we also report accuracy (1-misclassification error) and sensitivity and specificity of feature selection. The accuracy was calculated as the number of samples correctly classified in testing dataset divided by the total number of samples. The sensitivity (specificity) was calculated as the number of informative (uninformative) features correctly selected in the final model divided by the total number of informative (uninformative) features.

### Real data applications

#### Acute myeloid leukemia data

The Acute myeloid leukemia data for 344 samples were obtained from NCBI Gene Expression Omnibus, with accession numbers for gene expression and methylation data as GSE14468 (HOVON-SAKK cohort) and GSE18700, respectively. The data was also used and described by Taskensen et al. [14]. Briefly, for each patient sample, Affymetrix HGU133 plus2.0 (Santa Clara, CA, USA) and HELP-assay [24] was used to measure the gene expression and DNA methylation data, respectively. We filtered the gene features with fewer than 10 unique expression values, resulting in 46,083 gene features. All of the 25,626 DNA methylation features were considered in this study. Groups of AML patients that are characterized by common cytogenetic or molecular abnormality are denoted as subtypes. From the 15 subtypes studied by Taskesen et al. [14], we focus on the 7q AML subtype, characterized by partial or complete deletion of the genome fragments on the long arm of chromosome 7. In the 344 patients, 35 have 7q AML, and the rest can be characterized as non-7q. The multi-tuning parameter Elastic net was used to build classifiers by combining gene expression and DNA methylation data to differentiate 7q AML from the rest of the subtypes.

#### Prostate cancer

The prostate adenocarcinoma data was obtained from the Genomic Data Commons (GDC) Data Portal (Project ID TCGA-PRAD) and assembled by our collaborators at USC. In total 444 samples with both RNA-Seq gene expression and HumanMethylation450 (HM450) array data available were considered. The goal is to classify tumor aggressiveness, defined by both grade and stage of prostate cancer. We defined the tumor samples with Gleason score 7 and below, and T category of T1 or T2 (T2a, T2b and T2c) as non-aggressive, and those with Gleason score 7 or higher and T category of T3 (T3a, T3b) and T4 as aggressive. This resulted in 143 samples defined as non-aggressive and 265 defined as aggressive. Thirty-six samples (8%) were omitted for being high grade/low stage (25) or low grade/high stage (11) and unknown aggressiveness. After filtering gene features with zero variance across all samples 20,216 gene features remained. For the HM450 DNA methylation array, we excluded probes targeting SNPs, mapped to the X and Y chromosomes, in cross-reactive regions, and having missing values in at least one sample. This resulted in 371,513 remaining features. As customary, the gene expression data were log2 transformed to reduce the skewness of the gene expression distribution in its original scale.

#### Data analysis

We split the data into training and test sets to build a prediction model and assess its performance. Training was performed using a random 80% of the samples, stratified by outcome to preserve the original proportion of positive and negative cases in the full data. The remaining 20% of samples were used to compute the test AUC. In order to obtain stable estimates, the data splitting, model training and testing was repeated 50 times with the average performance from the independent test sets reported. In the training step, MTP EN penalties were tuned by 5-fold cross-validation over a grid of values ranging from 0.1 to 1.5 for the penalty ratio parameter *κ*, and a large grid of values for the overall penalty parameter *λ*. The 5-fold CV was repeated 10 times and the optimal *λ* for each ratio value *κ* was selected as the one yielding the largest mean AUC across the 10 CV repeats.

## Results

### Simulation studies

**Fig. 1:**
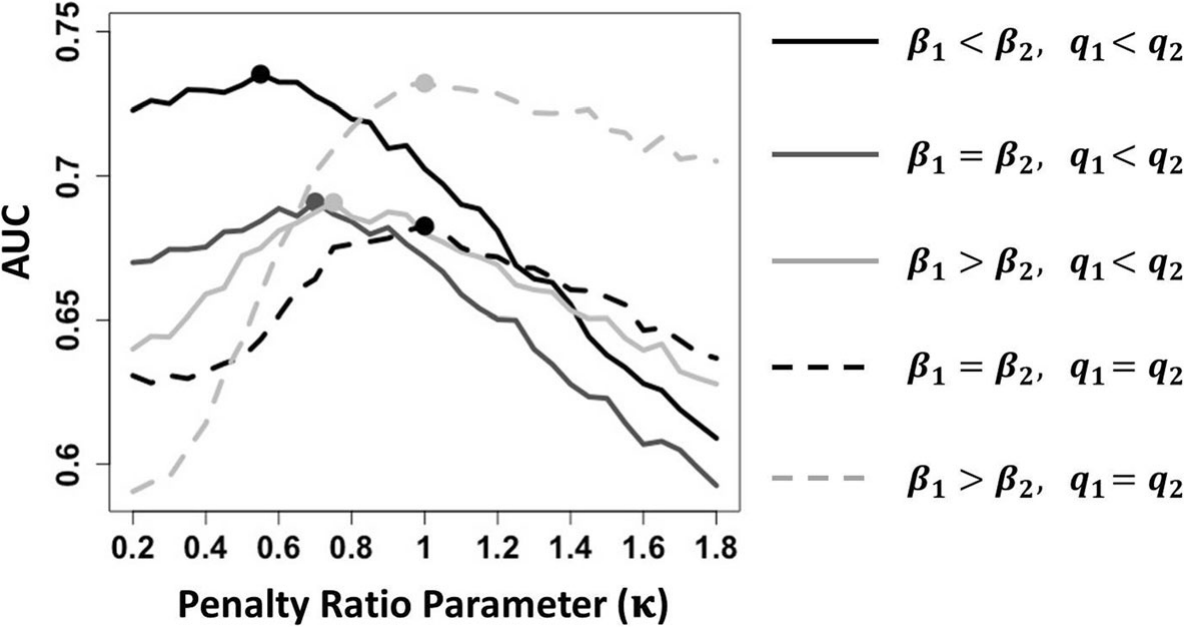
Mean testing AUC as a function of the penalty ratio parameter *κ* for different simulation settings. The effect sizes and numbers of informative features are given in Table 1, Scenarios 1–5. Dots indicate the *κ* resulting in the maximum of mean testing AUC. Each analysis includes 200 samples per data set with 250 features per data type (*N* = 200 simulation replicates). When the number of informative features differs among the platforms (Scenarios 1–3), the multi-tuning parameter EN yields more predictive models comparing with the standard EN where *κ*=1. Differential penalization increased AUC the most when the effect sizes are smaller in the omic type with fewer informative features (Scenario 1)

The main finding (see Figure 1) is that when the number of informative features differs between the two omic types (*q*_1_ < *q*_2_), differential penalization can yield a model with better prediction performance in terms of AUC. Specifically, the optimal parameter tuning favors a larger penalty on the omic type with fewer informative features (*κ* < 1) (Table 1, scenarios 1–3). Differential penalization increased AUC the most when the effect sizes are smaller in the omic type with fewer informative features (Figure 1). By contrast, when the two omic types have the same number of informative features (*q*_1_ = *q*_2_), the optimal penalty ratio parameter *κ* is close to 1, indicating that the standard EN yields the best predictive performance. For Scenarios 1–3 we also evaluated the performance of MTP-EN in terms of accuracy and sensitivity and specificity of feature selection (Additional file 2). Consistent with Figure 1, MTP-EN achieves higher rates of correct classification and better sensitivity in Scenarios 1–3. In terms of specificity, MTP-EN outperforms standard EN in Scenario 1. In Scenario 2 and 3, however, standard EN shows slightly higher specificity.

**Fig. 2:**
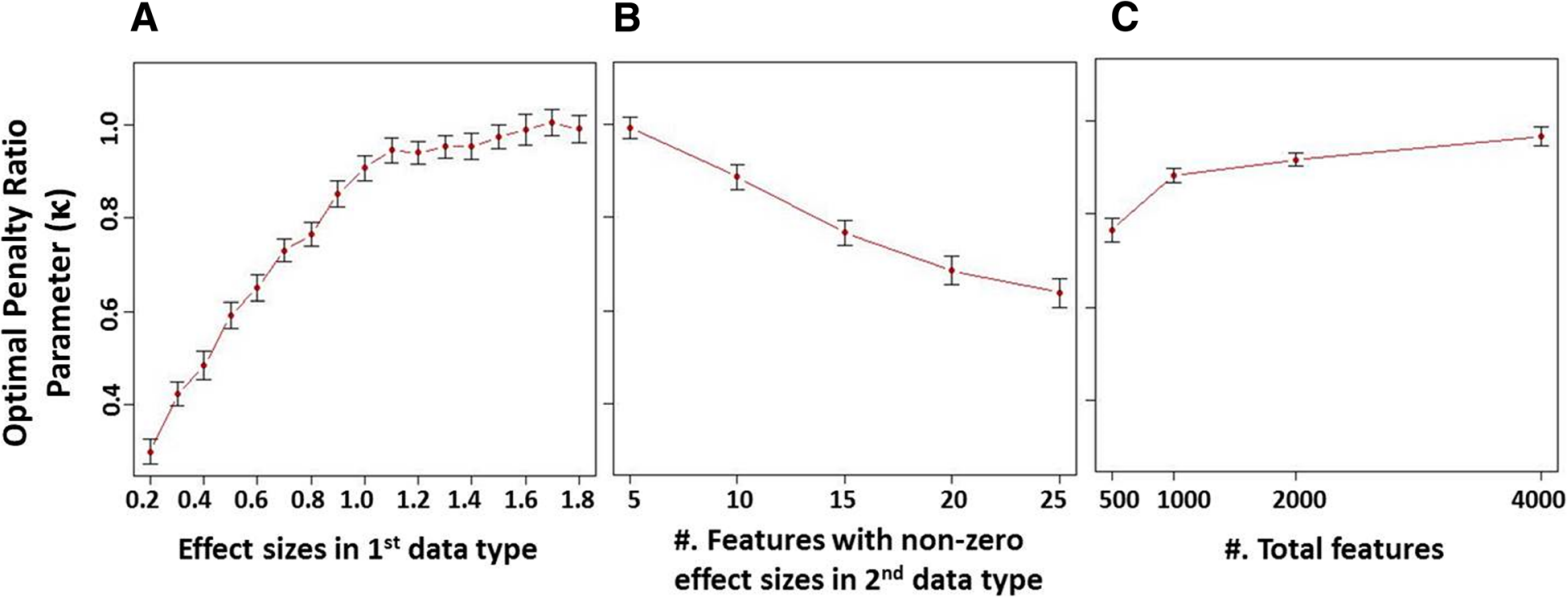
Factors associated with the change of optimal penalty ratio parameter *κ*. **a** For the scenario with different numbers of informative features, *q*_1_ < *q*_2_, *κ* increases monotonically to 1 (i.e. less differential penalization) as the effect size in the first omic type increases. **b** For fixed effect sizes *β*_1_ and *β*_2_, *κ* becomes smaller (more differential penalization) as the number of informative features in the second omic type increases. **c** As the overall proportion of informative features of both types, $\frac{q_1+{q}_2}{p}$, decreases relative to the total number of features, *κ* approaches 1, i.e. less differential penalization is required to maximize the AUC. Dots represent the optimal weights and caps represent the standard error of the mean; N = 200 simulated data sets

We explored the change of the optimal penalty ratio parameter as we varied characteristics of the true regression model one at a time. For the scenario with different numbers of informative features, *q*_1_ < *q*_2_, the optimal penalty ratio parameter *κ* is smallest (i.e. there is maximal differential penalization) when the effect sizes are smaller in the omic type with fewer informative features (*β*_1_ < *β*_2_), and it increases monotonically to 1 (i.e. less differential penalization) as the effect size, *β*_1_, in the first omic type increases (Figure 2a). For fixed effect sizes *β*_1_ and *β*_2_, the optimal penalty ratio parameter *κ* becomes smaller (more differential penalization) as the number of informative features in the second omic type, *q*_2_, increases (Figure 2b). In this case, it is advantageous to penalize the omic with more noise features more highly. As the overall proportion of informative features of both types, $\frac{q_1+{q}_2}{p}$, decreases relative to the total number of features, the optimal penalty ratio parameter *κ* approaches 1, i.e. less differential penalization is required to maximize the AUC (Figure 2c). With the decrease of $\frac{q_1+{q}_2}{p}$, the AUC for both the standard and MTP EN decreases and also the difference in AUC between the two approaches decreases (results not shown).

Because correlation among features both within- and between- omic types is common, we also investigated the effects of correlations on the performance of MTP EN. Across all settings we fixed the effect sizes and number of informative features as described in the third scenario in Figure 1 (*β*_1_ > *β*_2_, *q*_1_ < *q*_2_). We compare the situations where the correlations are only between informative features within the individual platforms (Table 1, scenario 6), and correlations both within- and between- platforms (scenario 7). We find that the higher the correlations between informative features, the higher the AUC for a given weight parameter, and the closer the optimal weight is to 1. This can be explained by the fact that the higher correlations increase the chance the set of correlated informative features are selected by standard EN, improving prediction performance without requiring the help of weight parameters. As a matter of fact, the advantage of MTP EN comparing with the standard EN is more obvious when there are fewer correlated features (Additional file 3A), or the correlation coefficients are smaller (Additional file 3B and C). Further, the correlations between the informative features from different platforms have an even larger impact on the performance of MTP EN than the correlations between the features from a single platform (Additional file 3B and C).

### Real data analysis

**Fig. 3:**
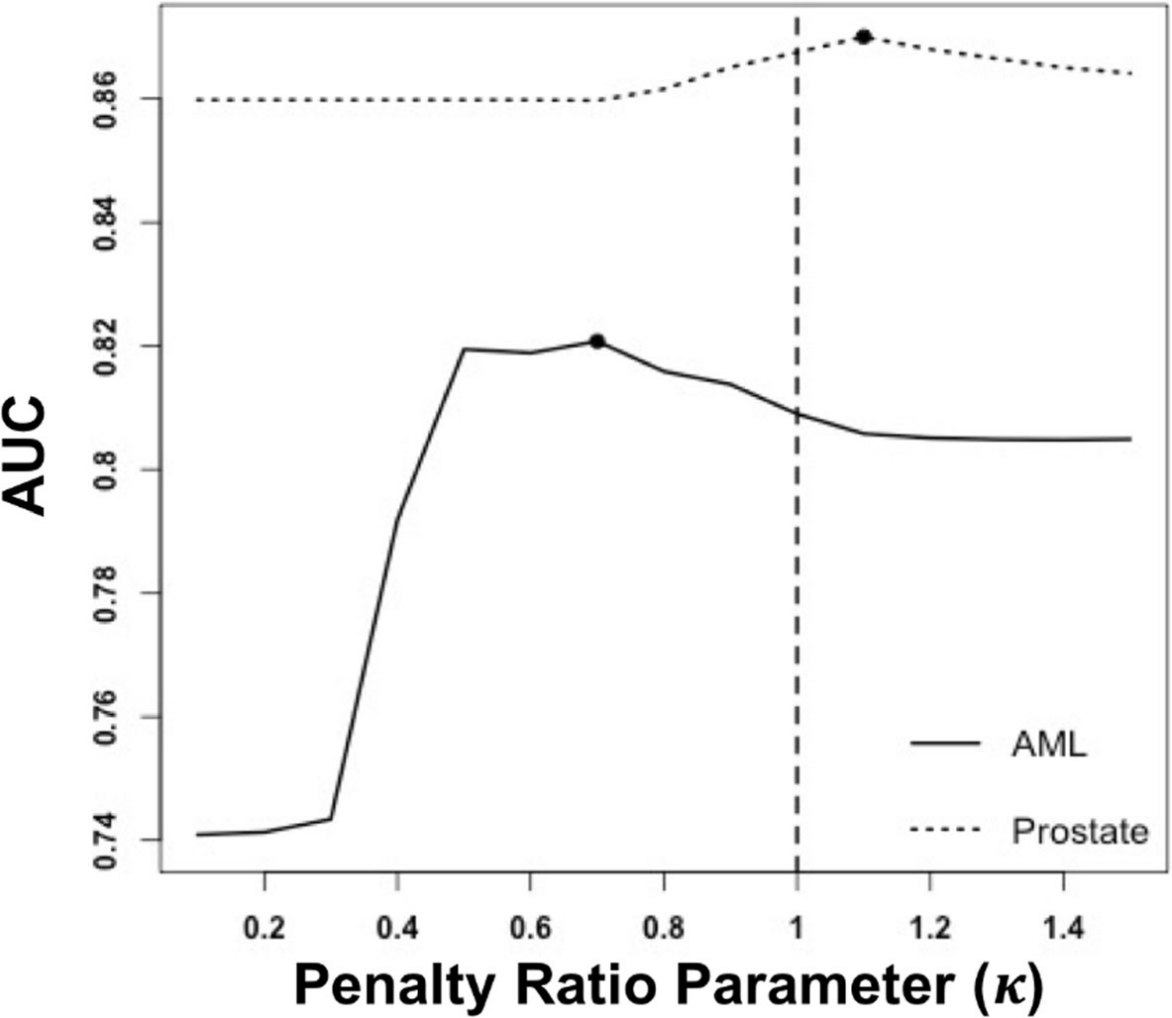
The AUC as a function of the penalty ratio parameters *κ* in cancer data sets. Solid line: AML data set; dashed line: Prostate cancer data set. The dark dots represent the *κ* that resulted in the maximum of mean test set AUC. For the AML data, a better classifier is obtained by adding relatively less penalty on the methylation features. For the prostate cancer data, there was very little difference in prediction performance between using data from a single platform or combining it

We also applied the MTP EN to two real cancer data sets, combining gene expression and DNA methylation data for outcome prediction. For the AML data, the goal is to discriminate the 7q subtype from the rest. Classifying patients as 7q is clinical significance as the subtype is characterized susceptibility to infection, quick aggravation, treatment resistance and poor prognosis [25, 26]. We found that the MTP EN achieved the best performance at the weight of 0.7 (solid line in Figure 3), indicating that by penalizing less the methylation features, we can obtain a better classifier than using standard elastic-net. This is consistent with previous finding that the molecular subtypes involving chromosomal abnormalities such as − 7/7q- could not be correctly predicted using gene expression profiling alone [27], while DNA methylation signatures have been shown predictive in classifying 7q subtype [28, 29]. Using MTP-EN we have a better chance to keep informative methylation features in the final model, which in turn yields improved prediction performance over using a standard single EN penalty regression.

For the prostate cancer data, the goal is to classify tumor aggressiveness, defined by both grade and stage of prostate cancer. In contrast to the AML data, in prostate cancer data the MTP EN achieved the best performance at the weight of 1.1 (dashed line in Figure 3), indicating MTP-EN yields almost equivalent prediction performance over standard EN. In fact, either DNA methylation or gene expression data alone achieve good prediction, which is consistent with previous findings [30, 31]. Given that the two platforms have similar prediction performance, the observation of little difference between MTP-EN and standard EN is within expectation.

We also investigated the difference in feature selection between MTP EN and standard EN for the two cancer data sets. Differential feature selection was defined by the numbers of times a variable was selected across the 50 repetitions of cross-validation. Since the optimal weight parameter in AML data is less than 1, we expect the MTP EN model to select more methylation features than the standard EN. Indeed, six methylation loci, seldom selected by standard EN, were often selected by MTP EN. Furthermore, the majority of those six features are associated with blood cancer (Additional file 4). Moreover, the genes that were selected by standard EN were also selected by MTP EN. In the PRAD data the opposite occurred. The optimal weight parameter was slightly larger than 1 and MTP EN selected more gene expression features than standard EN (Additional file 4). Four of the five genes, which were not selected by standard EN, have previously been identified as associated with cancer.

## Discussion

We propose a multi-tuning parameter elastic net (MTP EN) for the classification of samples with data from multiple –omic platforms. The MTP EN yields more predictive models in several scenarios, including when the proportion of predictive features is larger in one omic type. In all other scenarios, the predictive performance of MTP EN matches that of the standard single penalty EN, so there is no performance downside for using MTP EN. Importantly, MTP EN can be fitted using standard EN software like the ‘glmnet’ R package.

However, the MTP EN requires the tuning of one penalty parameter per omic type, and the computational effort for penalty-parameter-tuning using cross-validation grows exponentially in the number of tuning parameters when using a grid search. This should not prove a limitation for the majority of current studies that collect data on a few omic types only. Alternatively, much less costly parameter tuning using a random search of the tuning parameter space has been shown to be effective [32].

As is the case with penalized regression approaches in general, the benefit of MTP EN decreases with the increase of the number of noise features, so it is important to consider feature pre-selection before applying MTP EN. Excluding noise features and appropriately reducing dimensionality can maximize the performance of MTP EN.

The acute myeloid leukemia (AML) data set we used to illustrate the MTP EN has been previously analyzed by Taskesen et al. [14] using logistic regression with Lasso regularization to predict AML subtypes in 344 samples. They evaluated three different classification strategies, including early, late and no integration. In early integration, they combined gene expression profile (GEP) and DNA methylation profile (DMP) features first and then applied Lasso regression on all features to predict AML subtypes (‘concatenation-based’ integration). In late integration, they first used Lasso regression on GEP and DMP individually, and then trained a nearest mean classifier with the posterior probabilities of the GEP and DMP logistic regression as predictors (a two-layer classifier). They showed that early integration improved the predictive power compared to classifiers trained on GEP or DMP alone, and that in turn late integration outperformed early integration. Our results are consistent with Taskesen et al. regarding the performance of combined data versus the individual data; we observed that the maximum AUC was obtained at the weight of 0.7 (combined data) rather using methylation data only (equivalent to using a small weight in the MTP EN; left tail of the solid line in Figure 3) or using gene expression data only (equivalent to using a large weight; right tail of the solid line in Figure 3**).** More importantly, however, we noticed that the mean AUC for MTP EN is 0.82 for the optimal weight parameter, which is higher than the AUC of 0.80 obtained from the best method in Taskesen et al. These results further support the view that when we consider the inter-correlations between different platforms when setting up the prediction model, we can better utilize the complementary information across different platforms and obtain a model with better prediction performance.

We also applied our method on prostate adenocarcinoma (PRAD) TCGA data, which utilized the HM450 DNA methylation array and contained many more features than the AML data set. The change of the mean testing AUCs with the change of weight parameter was very slight in the PRAD data. Still, we identified several genes that had a much larger chance to be selected in MTP EN (under the weight parameter of 1.1) than in standard EN (weight = 1). Specifically, *ABCC5* has been reported to support osteoclast formation and promote breast cancer metastasis to bone [33], *ITGA11* has been identified to regulate cancer stromal stiffness and promote metastasis in non-small cell lung cancer [34], and *ZNF706* has been associated with tumor progression in head and neck cancer [35].

Although we only investigated the simple convex EN penalty, using multiple penalties is likely to be beneficial for more sophisticated convex penalties such as group- and fused-LASSO penalties but it remains an open question whether this would extend to non-convex penalties such as SCAD, or the minimax concave penalty [36].

## Conclusions

We proposed a multi-tuning parameter elastic net (MTP EN) model for the classification of samples with data from multiple –omic platforms, with separate tuning parameters for each omic type that can be fitted using existing software. We found that MTP EN yields a more predictive model than ordinary EN where a single penalty parameter is used for all features in different platforms, particularly when the proportion of informative features differs between platforms and when there is no notable correlation between the informative features.

## Additional files

Additional file 1: Self-contained R script for MTP-EN with full example. (R 5 kb)

Additional file 2: The accuracy in testing dataset, sensitivity and specificity of feature selection from Standard EN and MTP-EN for different simulation settings. MTP-EN achieves better classification and sensitivity in Scenarios 1–3. (PNG 214 kb)

Additional file 3: The optimal penalty ratio parameters versus the change of (A) number of correlated features in the second data type; (B) correlations among features in the second data type; and (C) correlations among features between different platforms. Dots represent the mean of optimal weights and caps represent the standard error of the mean; *N* = 200 simulation replicates. (PNG 100 kb)

Additional file 4: Listing of features that have more chance to be selected by MTP EN in AML and PRAD datasets. (DOCX 29 kb)

## Abbreviations

AML: Acute myeloid leukemia
CV: Cross-validation
DMP: DNA methylation profile
EN: Elastic net regression
GDC: Genomic Data Commons
GEO: Gene Expression Omnibus
GEP: Gene expression profile
HM450: HumanMethylation450
MTP EN: Multi-tuning parameter elastic net regression
PRAD: Prostate adenocarcinoma
TCGA: The Cancer Genome Atlas
