# Supplementary figures and images for: Data integration by multi-tuning parameter elastic net regression
Source: BMC Bioinformatics. 2018 Oct 10;19:369. doi: 10.1186/s12859-018-2401-1 (PMC6180486; doi:10.1186/s12859-018-2401-1)

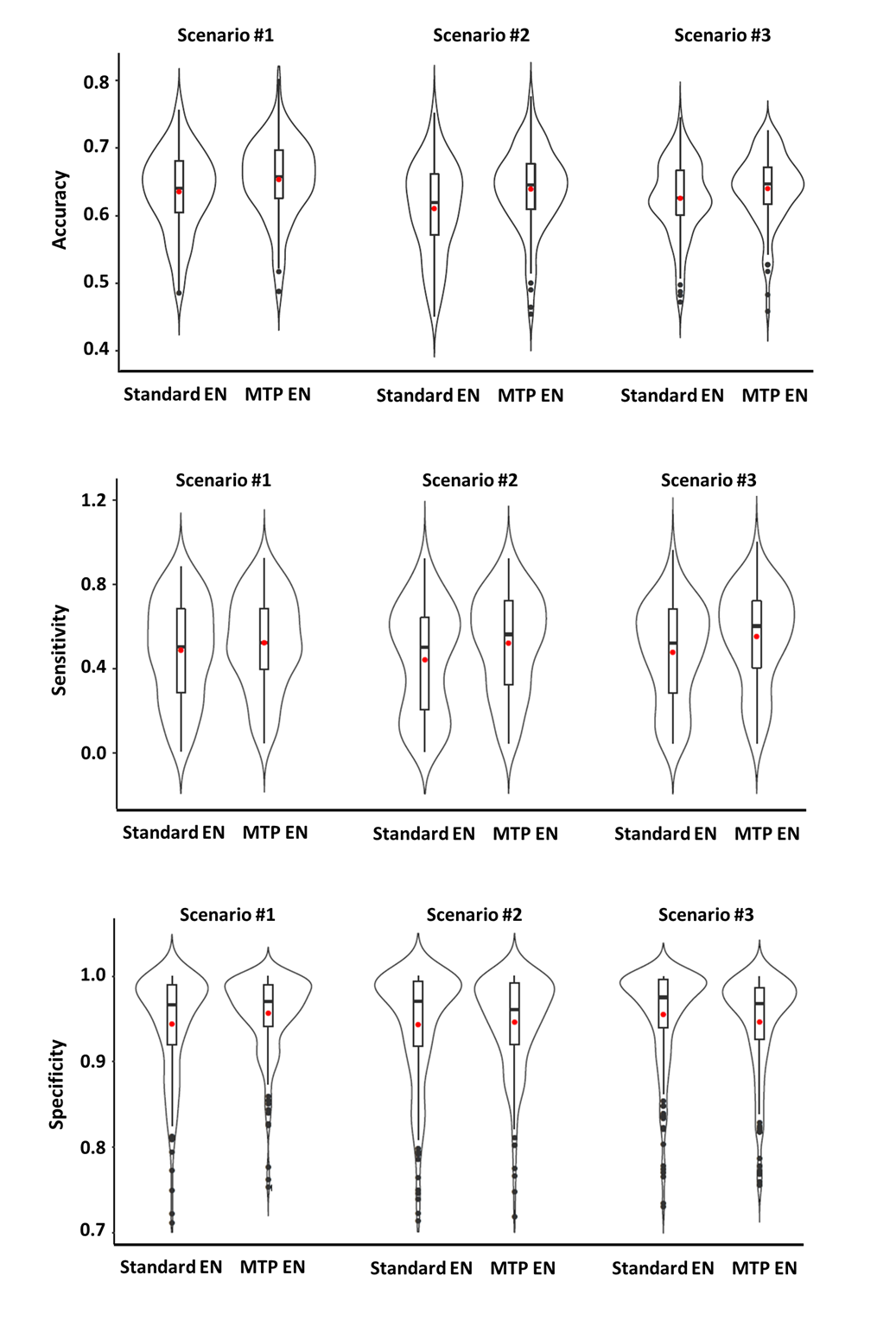

Supplement: Supplementary file 1 — Self-contained R script for MTP-EN with full example. (R 5 kb) [file 12859_2018_2401_MOESM1_ESM.png]

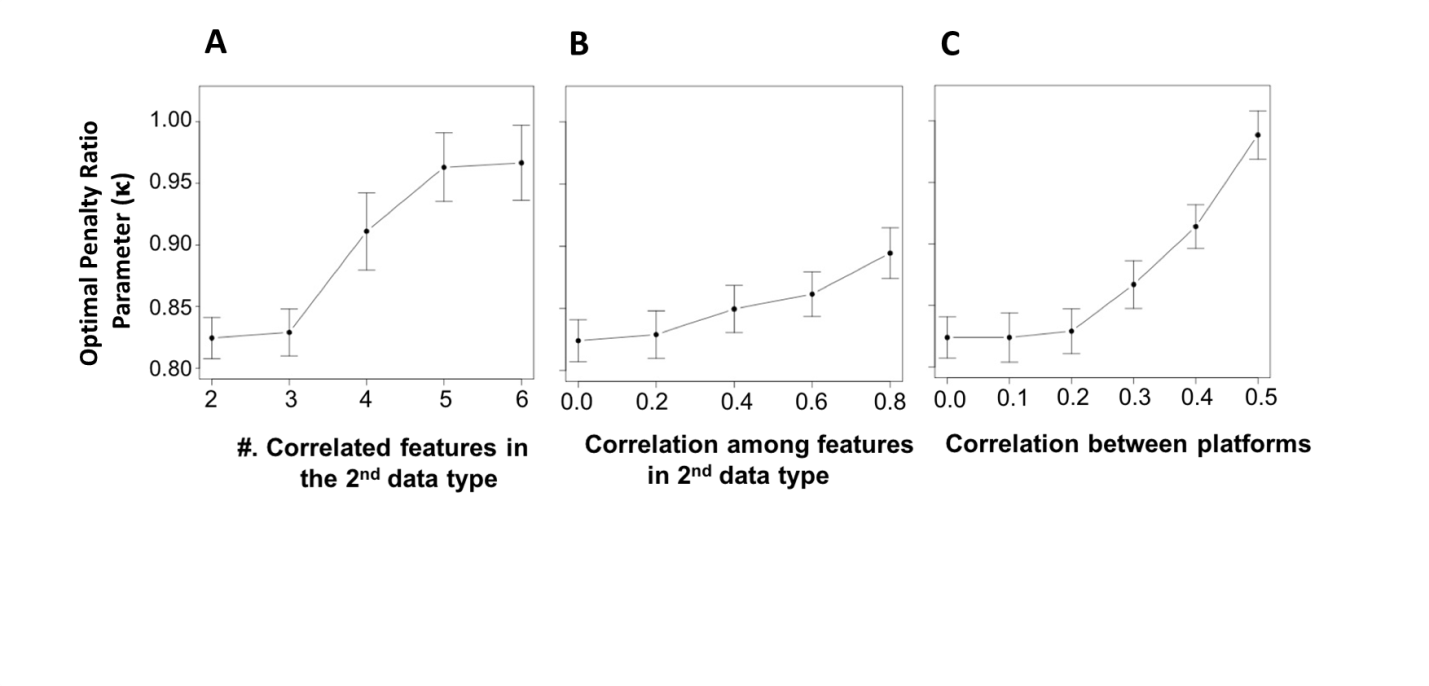

Supplement: Supplementary file 2 — The accuracy in testing dataset, sensitivity and specificity of feature selection from Standard EN and MTP-EN for different simulation settings. MTP-EN achieves better classification and sensitivity in Scenarios 1–3. (PNG 214 kb) [file 12859_2018_2401_MOESM2_ESM.png]
