# Supplementary material for: Data integration by multi-tuning parameter elastic net regression
Source: BMC Bioinformatics. 2018 Oct 10;19:369. doi: 10.1186/s12859-018-2401-1 (PMC6180486; doi:10.1186/s12859-018-2401-1)
Supplement: Supplementary file 3 — The optimal penalty ratio parameters versus the change of (A) number of correlated features in the second data type; (B) correlations among features in the second data type; and (C) correlations among features between different platforms. Dots represent the mean of optimal weights and caps represent the standard error of the mean; N = 200 simulation replicates. (PNG 100 kb) [file 12859_2018_2401_MOESM3_ESM.docx]

| ***DNA Methylation features that have more chance to be selected by MTP EN in AML data*** | | | | | |
| --- | --- | --- | --- | --- | --- |
| **MSIP ID** | **Gene ID** | **Chromosome** | **Gene Description** | **Associated Cancer** | **Ref** |
| MSPI0406S00140608 | WIPF1 | Chr2 | WAS/WASL Interacting Protein Family Member 1 | Leukemia and lymphoma | [28] |
| MSPI0406S00169876 | RARB | Chr3 | Retinoic acid receptor, beta | Myeloma | [29] |
| MSPI0406S00362230 | NPY | Chr7 | Neuropeptide Y |  |  |
| MSPI0406S00826420 | TRIM47 | Chr17 | Tripartite Motif Containing 47 | Burkitt lymphoma | [30] |
| MSPI0406S00871873 | SLC44A2 | Chr19 | Solute Carrier Family 44 Member 2 |  |  |
| MSPI0406S00403273 | RHEB | Chr7 | Ras Homolog Enriched in Brain | Associated with tumorigenesis | [31] |

| ***Genes that have more chance to be selected by MTP EN in PRAD data*** | | | | |
| --- | --- | --- | --- | --- |
| **Gene ID** | **Chromosome** | **Gene Description** | **Associated Cancer** | **Ref** |
| ABCC5 | Chr3 | ATP Binding Cassette Subfamily C Member 5 | Prostate Cancer | [32,33] |
| FAM220 | Chr7 | Family with Sequence Similarity 220 Member A |  |  |
| IGSF1 | ChrX | Immunoglobulin Superfamily Member 1 | Pituitary tumor | [34] |
| ITGA11 | Chr15 | Integrin Subunit Alpha 11 | Non-small cell lung cancer | [35,36] |
| ZNF706 | Chr8 | Zinc Finger Protein 706 | Prostate cancer | [37] |
